# Exploring the binding interaction of rupatadine with bovine serum albumin using multi-spectroscopic and molecular modeling approaches

**DOI:** 10.1186/s13065-026-01769-2

**Published:** 2026-03-28

**Authors:** Heba Abo Shamiya, Heba Elmansi, Shahenda M. El-Messery, Fathalla Belal

**Affiliations:** 1 Pharmaceutical Analytical Chemistry Department, Faculty of Pharmacy, Mansoura University, Mansoura, 35516 Egypt; 2 Pharmaceutical Organic Chemistry Department, Faculty of Pharmacy, Mansoura University, Mansoura, 35516 Egypt

**Keywords:** Rupatadine, Fluorescence, Bovine serum albumin, Thermodynamic, Docking

## Abstract

Rupatadine (RUPA), a second-generation H_1_-receptor antagonist, is used to treat allergies with a further antagonistic action on platelet-activating factor. Here, the binding interaction between RUPA and bovine serum albumin (BSA) has been investigated via various approaches, including spectrofluorimetric techniques, thermodynamic studies, Fourier transform infrared spectroscopy (FTIR), ultraviolet (UV) spectroscopy, and molecular docking (MD). The spectrofluorimetric titration study was displayed at various temperatures, and the data revealed that the BSA native fluorescence is quenched by RUPA via a static quenching process, which has been signified by UV absorption and fluorescence spectroscopy. The thermodynamic analysis revealed that the stoichiometry between RUPA and BSA is 1:1, and their binding affinity was weak to moderate. As revealed by the enthalpy change (ΔH) and entropy change (ΔS) values of 133.024 kJ mol^− 1^ and 0.487 kJ mol^− 1^, respectively. The hydrophobic forces are the main binding forces in the interaction between BSA and RUPA. The negative values of Gibbs free energy change (ΔG) indicate that the binding process between RUPA and BSA was spontaneous. Furthermore, results of the site marker technique and synchronous fluorescence measurements indicate that RUPA binding interaction occurs at site (I) on BSA in the vicinity of tryptophan residues, which was then confirmed by MD.

**Supplementary Information:**

The online version contains supplementary material available at 10.1186/s13065-026-01769-2.

## Introduction

Investigation of drug-protein binding interactions helps in the discovery of protein binding sites for targeted therapies, and its evaluation has been essential for the development of innovative drugs as well as the advancement of knowledge regarding drug action mechanisms and metabolic processes [1]. The characteristics of the plasma protein albumin, such as its high binding capabilities for both hydrophobic and hydrophilic medicines, comparatively long half-life, ability to specifically target inflammatory sites, and essentially virtually low toxicity and immunogenicity, have drawn particular attention to it [2].

Serum albumin is the predominant circulating protein in blood plasma, contributing to its good biocompatibility, biodegradability, and safety for therapeutic use [3]. Its chemical structure and conformation enable interaction with a variety of medicines, which may shield them from metabolism and excretion in vivo and enhance their pharmacokinetic characteristics [4].

Because of the high solubility, low cost, and structural homology between bovine serum albumin and human serum albumin (76%), it has been used extensively to investigate interactions between ligands and serum albumin. The BSA molecule consists of a single polypeptide chain of three homologous α-helical domains (I, II, III), with each domain including two subdomains (A and B) [5].

It is crucial to delve deeper into the characteristics of the drug-protein interactions through diverse approaches using BSA as a protein model to study such interactions in order to minimize side effects and maximize the effectiveness of the medicines. El Gammal et al. [6] used such approaches to study similar binding interactions in vitro under simulated physiological circumstances (pH 7.4) utilizing the quenching fluorescence technique, and the parameters of thermodynamics were applied in order to determine the binding constants at three different temperatures.

As well as synchronous fluorescence, Fourier transform infrared spectroscopy (FTIR), UV-visible spectroscopy, the site marker technique, and MD have been applied for exploring the site of interaction and the protein structure’s alterations upon binding with RUPA [7–11].

Here, we studied how RUPA binds to BSA in order to elucidate the interaction mechanism of the binding utilizing several fluorescence spectroscopy techniques, Fourier transform infrared spectroscopy, and UV-visible spectroscopy. Furthermore, a detailed MD analysis was used to determine the most preferred binding site within BSA.

**Fig. 1:**
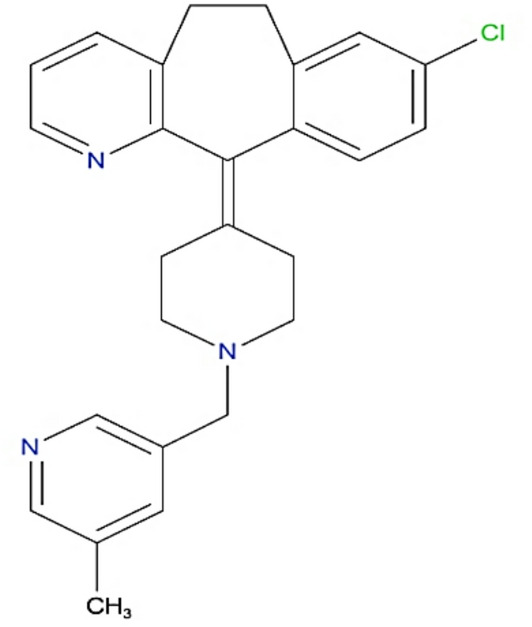
Structure of rupatadine

Rupatadine (Fig. 1) chemically is 13-chloro-2-[1-[(5-methylpyridin-3-yl) methyl] piperidin-4-ylidene]−4-azatricyclo [9.4.0.03,8] pentadeca-1(11),3(8),4,6,12,14-hexaene [12]. Rupatadine is a second-generation antihistamine and antagonist of platelet-activating factor utilized for allergy treatment. It was discovered, manufactured, and commercialized as Rupafin 10 mg and various other brand names in 2003. It is approximately 99% attached to plasma proteins, despite its extensive distribution to other tissues [13].

Deuster et al. [14] found in 2021 that RUPA targets the platelet-activating factor receptor to prevent ovarian cancer cells from proliferating and migrating in vitro. According to these results, RUPA could potentially have anticancer properties.

As far as we are aware, we have evaluated the behavior of the RUPA-BSA binding interaction for the first time. This evaluation assists in improving its pharmacological and clinical efficacy, ensuring effective therapeutic drug levels, and preventing any potential unwanted adverse events.

## Experimental procedure

### Chemicals

Bovine serum albumin (standard grade, pH 7, Origin USA) was purchased from Cegrogen Biotech GmbH (Germany). The molar concentration of BSA was calculated using the assumption that its molecular weight was 66,500 Da. Rupatadine fumarate was kindly supplied by ATCO PHARMA for Pharmaceutical Industries (Cairo, Egypt). Diazepam (99.2% purity) was obtained from EIPICO (10th of Ramadan City, Egypt). Phenylbutazone (99.7% purity) was obtained from GLOBAL NAPI PHARMACEUTICALS (6th October City, Egypt). Tris hydrochloride (Tris–HCl) batch number 337,012,022 (purity > 99%) was purchased from Chemajet for Chemical and Pharmaceutical Industries, Alexandria, Egypt.

The supplier of HPLC-grade methanol was Sigma Aldrich (St. Louis, MO, USA). Double-distilled water was utilized. All chemicals used were of analytical reagent-grade.

### Instrumentation

Agilent Technologies’ Cary Eclipse fluorescence spectrophotometer (Santa Clara, California, USA) was utilized. The quartz cuvette utilized was 1 cm. The slit width was 5 nm, and the smoothing factor was 19. The wavelengths were 279 and 341 nm, as excitation and emission, respectively. The voltage was adjusted to 600 V. Shimadzu UV-1601, a UV-visible double-beam spectrophotometer (Kyoto, Japan), was employed at a high scan speed. Quartz cuvettes with 1 cm path length were used for all measurements. A Bruker Tensor 27 FTIR spectrometer was used to scan the FTIR spectra under strictly constant conditions in the region of 400–5000 cm^− 1^. All pH readings were taken using a Jenway 3510 pH meter (Jenway, Staffordshire, UK).

### Software

The software “Molecular Operating Environment (MOE) 2024.06 was utilized for both the docking of RUPA to examine how it binds and interacts with BSA and the surface mapping study of RUPA.

### Preparation of solutions

A stock solution of 200 µM bovine serum albumin was prepared using bidistilled water, and rupatadine (1000 µM) was freshly prepared using bidistilled water. The buffer solution (pH 7.4) of Tris-hydrochloride (20 mM) was freshly prepared using bidistilled water. Phenylbutazone and diazepam stock solutions (10 mM) were prepared in methanol. To prepare the working solutions, all stock solutions were diluted beforehand and stored at 277 K.

### Spectroscopic measurements

#### Fluorescence measurements

The fluorescence quenching of BSA by RUPA was examined at three different temperatures: 303, 310, and 318 K, along with wavelengths (300 to 500 nm) following excitation at 279 nm. The concentration of BSA remained constant at 2 µM, whereas the concentration of RUPA was gradually increased from 0 to 100 µM.

#### Synchronous fluorescence technique

With increasing concentrations of RUPA (0–100 µM), by adjusting the Δλ at 60 nm for tryptophan (Trp) and Δλ at 15 nm for tyrosine (Tyr) residues at 303 K, synchronous fluorescence spectra of BSA were scanned in the 200–350 nm region.

#### FT-IR spectroscopy

The FTIR spectra of the BSA solution (2 µM), both without and with RUPA (40 µM) in Tris-HCl buffer (20 mM) at pH 7.4, were scanned over the 400–5000 cm^− 1^ region.

#### UV-visible spectroscopic measurements

The ultraviolet absorption spectra of the BSA solution (2 µM) with different amounts of RUPA (0–100 µM) in Tris-HCl buffer (20 mM) at pH 7.4 were measured over the 190–500 nm region in contrast to a reference solution that included all relevant system components except for BSA. The experiments were carried out at 303 K.

### Competitive binding analysis using site probes

Phenylbutazone and diazepam are the site probes for sites Ⅰ and Ⅱ, respectively. The BSA and site probe concentrations in this experiment were maintained at 2 µM and 10 µM, respectively. Rupatadine concentrations ranged from 10 to 100 µM. After excitation at 279 nm, fluorescence intensity measurements were made at 341 nm. At 303 K, measurements were made. The binding constants (K_b_) of RUPA to diazepam-BSA, phenylbutazone-BSA, and BSA alone were compared.

### Influence of metal ions on the RUPA-BSA binding interaction

Bovine serum albumin and metal ion solutions were fixed at 2 µM (pH 7.4), and the concentration of RUPA was varied from 10 to 100 µM. Fluorescence intensity measurements were taken at 341 nm following excitation at 279 nm at 303 K. Comparisons were made between the equilibrium constants for RUPA binding (K_b_) to BSA with each metal ion present and the equilibrium constant for RUPA binding (K_b_) to BSA alone.

### Molecular docking and surface mapping

The binding behavior of BSA and RUPA was investigated using the Molecular Operating Environment (MOE) 2024.06. The structure of RUPA was initially drawn in the MOE. A conformational search was performed to obtain the most stable conformer using the systematic search method. The crystalline structure of bovine serum albumin was acquired from the Protein Data Bank (PDB) using the PDB code number 4f5s (http://www.rcsb.org). The docking procedure has been carried out between the binding pocket and the conformers with the lowest energy, ‘global minima’. Following preparation, both ligands and protein receptors were protonated, and the force field MMFF94X’s default parameters were used to reduce energy.

The MD parameters utilized in the experiment have been employed with Triangle Matcher as a default. Along with 30 conformation generations to match the binding groove, rescoring functions 1 and 2 were set to London dG and GBVI/WSA dG, respectively. For additional investigation and assessment of the relationship between RUPA and BSA, the MDB output file was produced.

Surface mapping of both the binding pocket and ligand was produced in a color-coded map where pink denotes a hydrogen bond, blue denotes a mild polar region, and green denotes a hydrophobic region [15–20].

## Results and discussion

### Examination of quenching mechanism

**Fig. 2:**
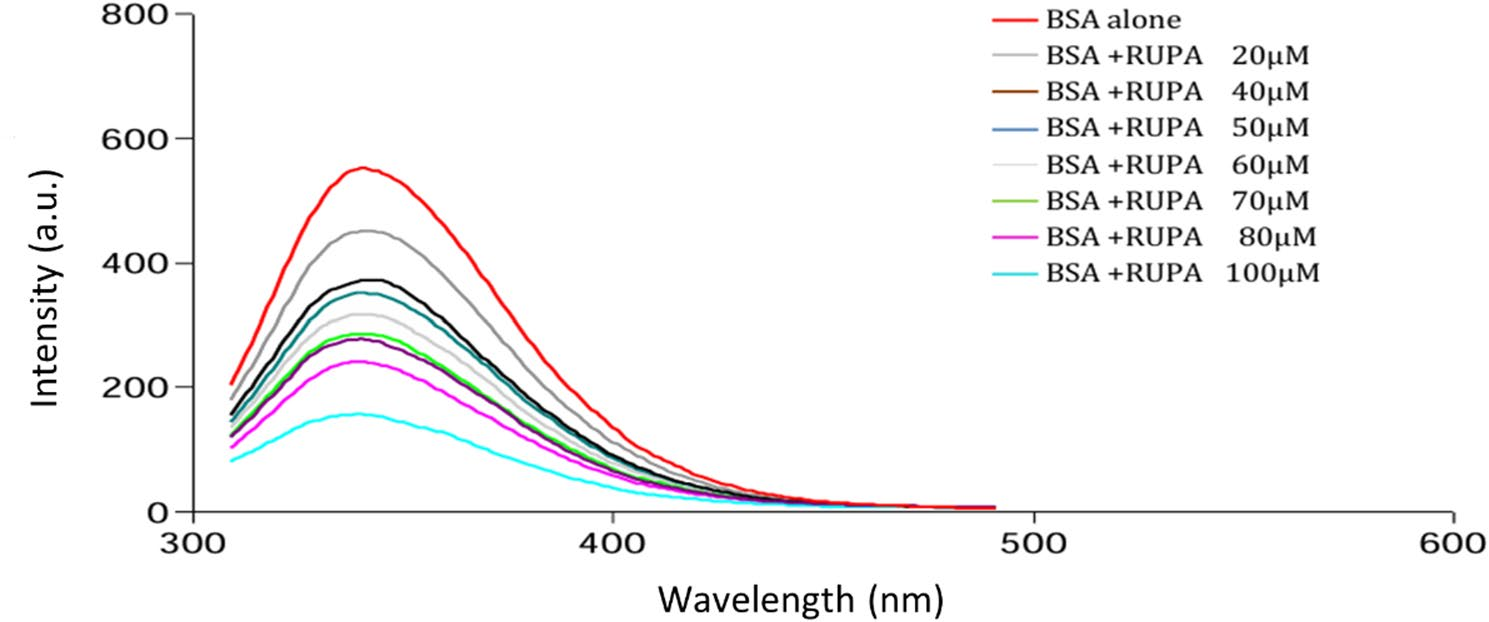
Fluorescence spectra of BSA (2µM) with growing amounts of RUPA from (0 to 100µM) at λ_ex_ = 279 nm and λ_em_ = 341 nm at 303 K

The presence of aromatic amino acid residues in the structure of BSA, such as tryptophan (Trp), tyrosine (Tyr), and phenylalanine (Phe), is what gives it its inherent fluorescence. Investigating protein conformational changes upon interactions with different drugs and providing precise information on ligand-protein interactions are two common uses for fluorescence spectroscopy [21]. In this experiment, we measured the decrease of the BSA intrinsic fluorescence due to quenching by RUPA (Fig. 2).

The fluorescence data were corrected for the inner filter effect (IFE) according to the Lakowicz method to ensure that the obtained binding parameters were not impacted by absorption artifacts [22].

The unavoidable dependence on approximations for certain factors and components in computations may lead to less-than-ideal modeling results. Measurements of RUPA absorbance at 279 nm (excitation wavelength) and 341 nm (emission wavelength) were made to determine the significance of IFE in this system. The absorbance values at 279 nm ranged from 0.08 (BSA alone) to 0.42 (BSA +100 µM RUPA), whereas at 341 nm, the absorbance was negligible. Consequently, it is essential to adjust the fluorescence intensity.

To account for the possible influence of inner filter effects (IFE), the fluorescence intensities were corrected using the following formula (Eq. 1) [22]:

1 $$F_{{corr}} = F_{{obs}} \times antilog^{{\left( {\frac{{Aex + Aem}}{2}} \right)}}$$

F_cor_ and F_obs_ are the corrected and measured fluorescence, respectively. A_ex_ and A_em_ are the absorption values at the excitation wavelength and emission wavelength, respectively.

Fluorescence quenching occurs through several mechanisms, including dynamic quenching, which arises from collisions between proteins and quenchers; static quenching, which results from ground-state complex formation between proteins and quenchers; and the combined mechanism that combines static and dynamic quenching, resulting in both complex formation and collision with the same quencher.

The process of fluorescence quenching was determined using the Stern-Volmer equation (Eq. 2):

2 $$F_{o} /F{\text{ }} = {\text{ }}1 + Kq{\text{ }}\tau o{\text{ }}\left[ Q \right]{\text{ }} = {\text{ }}1 + Ksv{\text{ }}\left[ Q \right]$$

F and F_0_ represent the emission intensity of BSA with and without RUPA, respectively. [Q] stands for the concentration of RUPA. K_q_, K_SV_, and τ_0_ denote the quenching rate constant, the Stern–Volmer quenching constant, and the fluorescence lifespan without RUPA, which is equivalent to 10^− 8^ seconds, respectively.

**Fig. 3:**
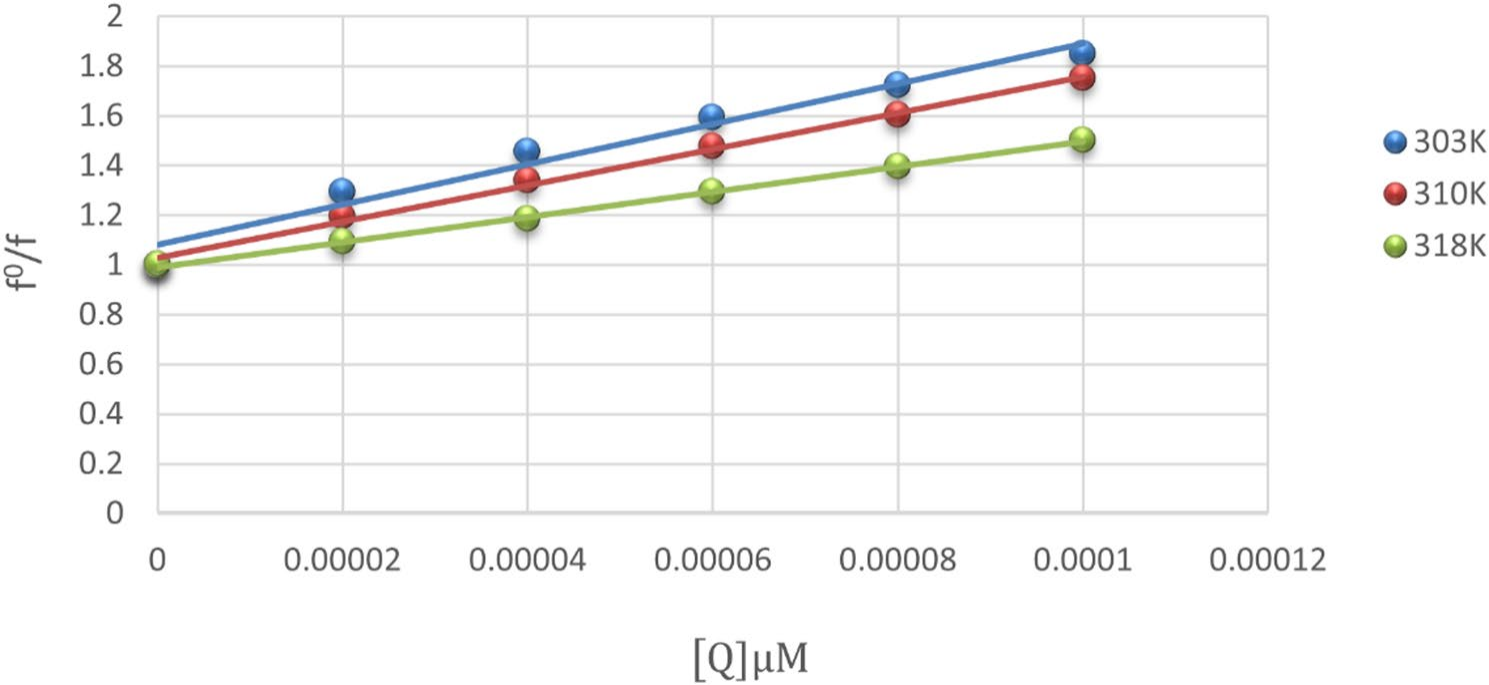
Stern-Volmer plot for the determination of quenching rate constant of RUPA at various temperatures

Generally, in the static quenching process, elevating the temperature causes the quenching constant to decrease, but in the dynamic mechanism, it induces an increase [23]. The K_SV_ and K_q_ values were determined by the graph of F_0_/F against [Q] (Fig. 3). Table 1 provides a summary of the K_SV_ and K_q_ values acquired at various temperatures. The data indicated that the K_SV_ value decreased with rising temperature in the presence of RUPA, and the k_q_ values exceeded the maximum K_q_ for dynamic (2 × 10^10^ L mol^− 1^ s^− 1^); hence, the quenching mechanism is a static process.

**Table 1:** RUPA-BSA interaction parameters at various temperatures

T(K)	K_sv_ (L.mol^− 1^)	K_q_ (L.mol^− 1^.s^− 1^)	*R* ^2^	SEM
303	1.07 × 10^4^	1.07 × 10^12^	0.9975	0.111
310	0.82 × 10^4^	0.82 × 10^12^	0.9994	0.042
318	0.48 × 10^4^	0.48 × 10^12^	0.9991	0.010

*SEM standard error of mean

### Determinations of the binding constant and stoichiometric analysis

Stoichiometric analysis is used to define the various equilibria for a macromolecule’s binding of a ligand by binding sites (n) [24]. The number of binding sites (n) and the binding constant (K_b_) were calculated using the modified Stern–Volmer equation (Eq. 3).

3 $$Log{\text{ }}F_{o} - F/F{\text{ }} = {\text{ }}log{\text{ }}K_{b} + {\text{ }}n{\text{ }}log{\text{ }}\left[ Q \right]$$

Thus, by graphing log [(F_0_ – F)/F] against log [Q], as illustrated in (Figure S1), we can obtain the number of binding sites (n) and the binding constant (K_b_) for the interaction of RUPA and BSA. The values of log K_b_, and (n) as seen in Table 2, are obtained from the intercept and slope, respectively.

**Table 2:** RUPA-BSA complex binding characteristics at different temperatures

T (K)	Log K_b_	K_b_	*n*	*R* ^2^	SEM
303	2.55	0.35 × 10^3^	0.57	0.9993	0.002
310	3.18	1.53 × 10^3^	0.83	0.9991	0.003
318	3.95	8.85 × 10^3^	1.06	0.9991	0.004

*SEM standard error of mean

It was observed that upon rising temperatures, the binding constant (K_b_) increased; this can be interpreted as the quenching being due to hydrophobic interactions rather than ionic interactions, as will be revealed in the next study. The binding constant (Kb) of 10^3^ L mol^− 1^ showed a weak to moderate binding affinity. The stoichiometry for RUPA-BSA binding is 1:1, which was indicated by the value of (n), which was close to 1.

### Thermodynamic characteristics and nature of binding forces

**Fig. 4:**
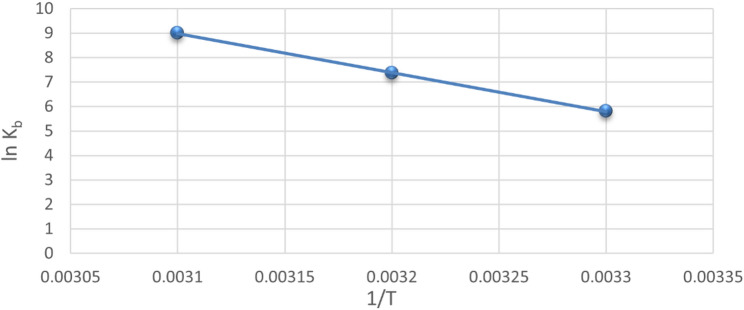
The van’t Hoff plot for BSA-RUPA binding

The thermodynamic characteristics of the binding reaction serve as the primary evidence for the binding force. Hydrogen bonds, hydrophobic forces, electrostatic interactions, van der Waals interactions, and other binding forces could all be present between small molecules and biomacromolecules [25]. The temperature dependence of the binding constant was thus measured for this purpose at three distinct temperatures: 303, 310, and 318 K (Fig. 4). Table 3 shows the thermodynamic parameters of the BSA-RUPA system, including enthalpy change (ΔH), entropy change (ΔS), and Gibbs free energy change (ΔG), which describe the binding forces. The values of ΔH and ΔS derived from the van’t Hoff equation (Eq. 4) are displayed in Fig. 4.

4 $$Ln{\text{ }}K_{b} = {\text{ }} - \Delta H/RT{\text{ }} + {\text{ }}\Delta S/R$$

The Gibbs–Helmholtz equation provided below was used to assess the free energy change (ΔG) value (Eq. 5).

5 $$\Delta G{\text{ }} = {\text{ }}\Delta H{\text{ }}{-}{\text{ }}T\Delta S$$

Within the examined temperature range, the values of the enthalpy change (ΔH) and the entropy change (∆S) were 133.024 kJ mol −1 and 0.487 kJ mol −1, respectively. Thus, hydrophobic interactions appear to represent the main binding forces in the interaction between RUPA and BSA, as indicated by the positive values of ∆H and ∆S. Furthermore, the positive value of ΔH signified that the creation of the RUPA-BSA complex was an endothermic reaction. The findings indicated that the binding interaction between BSA and RUPA was spontaneous because the ΔG values were negative.

**Table 3:** Thermodynamic parameters of BSA-RUPA interaction at pH 7.4

T (K)	∆H^o^(KJ/mol)	∆G^o^(KJ/mol)	SEM	∆S^o^(KJ mol^− 1^ K^− 1^)	*R* ^2^
303	133.024	−14.570	2.148	0.487	0.9998
310		−19.441			
318		−21.877			

*SEM standard error of mean

### Spectroscopic study of RUPA-BSA interaction

#### Synchronous fluorescence measurements

Using synchronous fluorescence, the influence of the ligand on the protein has been thoroughly investigated [26]. The distinctive details regarding the protein’s Tyr and Trp residues can be gained by fixing the scanning interval (∆λ) between the excitation and emission wavelengths at 15 and 60 nm, respectively. Figures S2 (a) and S2 (b) show the findings of the corresponding BSA 

#### UV-visible spectroscopic measurements

fluorescence ligand binding on the polarity of the microenvironment surrounding fluorescent amino acid residues of the protein spectra as RUPA concentrations rise. Adding RUPA and monitoring at ∆λ = 60 resulted in a minor red shift of ∆λ_max_ of about 2 nm, whereas no shift was observed at ∆λ = 15. This indicates that RUPA binds to BSA at Trp residues, as confirmed by MD experiments that revealed drug binding near Trp residues. For data validation, the spectral data were corrected for the inner filter effect in accordance with the previously described Lakowicz equation (Eq. 1) [22]. This indicates that RUPA binds to BSA at Trp residues, as confirmed by MD experiments that revealed drug binding near Trp residues.

#### FT-IR spectroscopy

FTIR measurements have been performed to further explore the BSA conformational change that is caused by the interaction with RUPA. The spectrum in Figure S3 (a) represents free BSA, and Figure S3 (b) is the spectrum of the RUPA-BSA complex at pH 7.4 at 303 K. The C = O stretch is responsible for the amide I band, which is located between 1600 and 1700 cm^− 1^ [27]. It is linked to the secondary structure of BSA and is more vulnerable to modifications in the secondary structure of proteins than amide II, which is located between 1500 and 1600 cm^− 1^. Following the RUPA reaction, the amide I peak’s location shifted from 1646 to 1642 cm^− 1^, indicating that secondary protein structure changes during the RUPA-BSA interaction.

#### UV-visible spectroscopic measurements

**Fig. 5:**
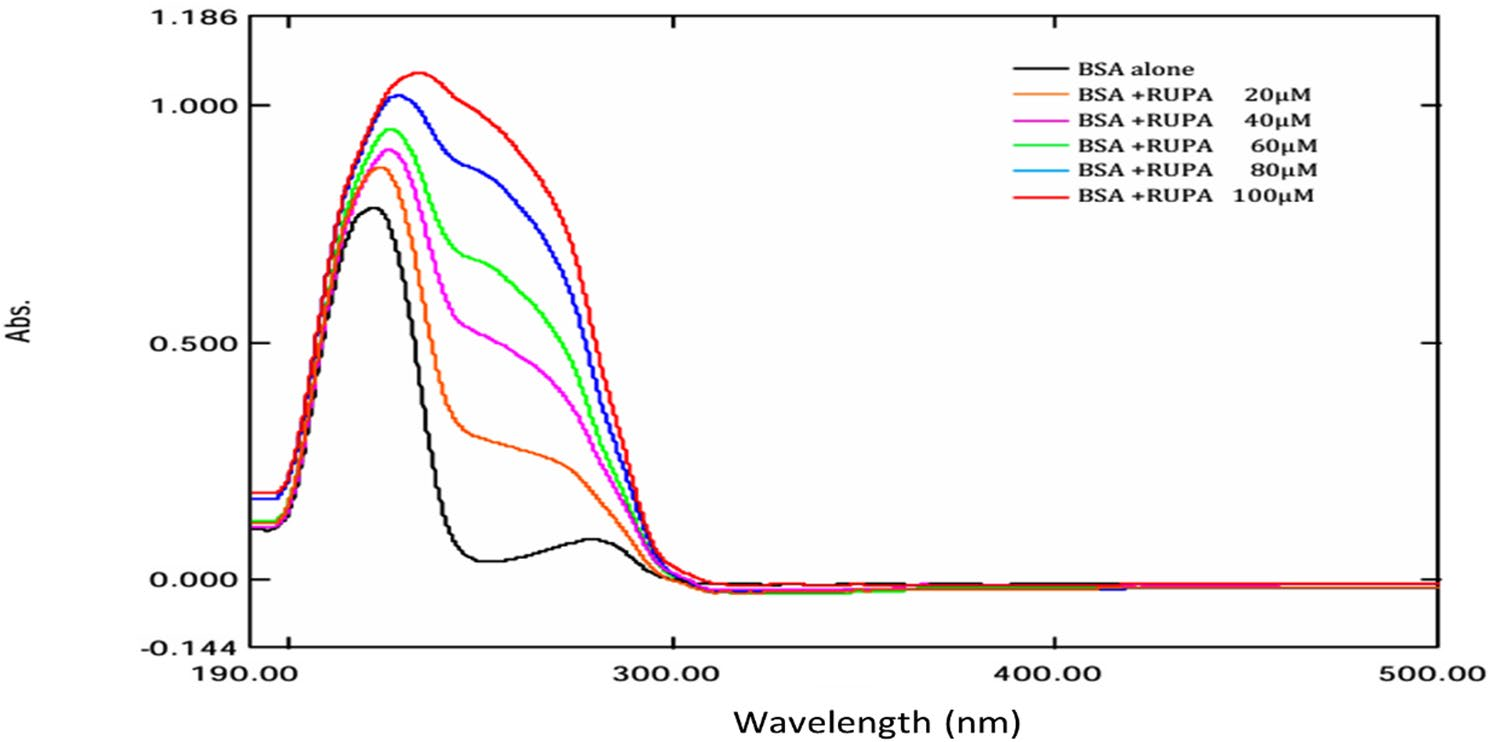
UV absorption spectra of BSA (2µM) with different concentrations of RUPA (0–100 µM) at pH 7.4 at 303 K

Ultraviolet-visible spectroscopy is an effective technique for identifying the conformational alterations of proteins mediated by drug molecules [28]. Alterations in the ultraviolet-visible spectra of the protein, whether in intensity or λ_max_, are primarily seen in the static process because a new ground-state complex has been established; conversely, dynamic quenching typically does not correlate with modifications in the protein’s absorbance spectrum [29, 30]. As shown in Fig. 5, the UV absorption spectrum of BSA was captured both without and with RUPA added; it can be found that there were two absorption bands for all solutions of BSA. The band of absorption close to 220 nm associated with the α-helix indicates the structural shape of bovine serum albumin. A modest absorption band at about 280 nm is attributed to the π–π* transitions of aromatic amino acid residues, including tryptophan, tyrosine, and phenylalanine. A notable escalation in intensity is observed with rising RUPA concentrations, accompanied by a red shift at the maximum wavelength near 220 nm. The UV-Vis absorption spectra have been calculated as molar absorptivity (ε, M⁻¹cm⁻¹) at λ_max_ = 279 nm for the binding mixture (BSA + RUPA, ε = 56,000 M⁻¹cm⁻¹) after correcting for inner filter effects (Fig. S8). This indicates the involvement of static quenching and corroborates the earlier findings of the fluorescence quenching investigation.

### Competitive binding analysis using site probes

Two specific site probes, phenylbutazone for site I and diazepam for site II, were used as site probes in site marker competitive binding experiments to identify the preferential binding site of RUPA on BSA.

For small ligands, BSA has two primary binding sites: site I, which is located in subdomain IIA’s hydrophobic pocket, and site II, which is located in subdomain IIIA’s hydrophobic cavity. Site I is comparatively bigger and mostly bound by hydrophobic interactions with neutral, bulky, and heterocyclic molecules. However, site II is smaller, and the interaction usually happens via a mix of electrostatic, hydrogen bonding, and hydrophobic forces [31–33]. The K_b_ value for RUPA binding with BSA in the presence of phenylbutazone was found to be lower than that in the absence of the site marker, as shown in Table 4. However, there was no discernible variation in the K_b_ value when diazepam was present. According to these findings, phenylbutazone and RUPA vie for the same binding site at BSA, which is site I.

**Table 4:** Binding constant of RUPA in the absence and presence of site markers at 303 K

Site marker	Log K_b_	*R* ^2^	SEM
Blank	2.55	0.9993	0.002
Phenylbutazone	1.91	0.9970	0.005
Diazepam	2.50	0.9984	0.003

*SEM standard error of mean

### Influence of metal ions on RUPA-BSA interaction

Metal ions can significantly influence protein-ligand interactions. Through the creation of coordination bonds, metal ions alter the biological function and structure of proteins; as a result, they have an impact on how the proteins interact with medications and how they bind. Such interactions can have direct implications for the pharmacokinetics of RUPA, as changes in protein binding may affect its distribution, bioavailability, and clearance [34, 35]. This work was thought to be valuable because proteins are utilized in a wide range of sectors and have a number of functions in the human body, where interactions with certain metals are significant. According to earlier research, depending on their nature and concentration, several metal ions can either increase or decrease ligand affinity. In order to better understand the binding mechanism of RUPA with BSA and its possible pharmacokinetic behavior, it is important to examine the impact of metal ions [36, 37]. For that, the influence of some metal ions (Na^+ 1^, K^+ 1^, Zn^+ 2^, Ca^+ 2^, Mg^+ 2^, Fe^+ 3^) on the BSA-RUPA system was explored. Monovalent ions such as Na⁺ exhibited lower K_b_, which may be a result of competitive binding with BSA. Conversely, K⁺ markedly increased the binding, which could be attributed to the formation of the ion-RUPA-BSA system, as shown in Table 5. The determined K_b_ values of the RUPA-BSA complex in the presence of divalent cations, such as Zn^+ 2^, Ca^+ 2^, and Mg^+ 2^, were greater than those without them. This may be explained by the formation of the metal ion-RUPA-BSA system. On the other hand, Fe³⁺ (a trivalent cation) exhibited minimal impact on the binding affinity of RUPA to BSA. The slight impact may be attributed to the high charge density and pronounced hydrolytic propensity of trivalent ions, which frequently induce nonspecific aggregation or weak electrostatic contacts instead of stable competition at the primary binding sites of BSA. However, the change of K_b_ in general could be attributed to changes in BSA confirmation by its interaction with metal ions at metal binding sites. Further research is needed to understand the type, strength, and role of a protein’s functional groups in metal-protein interactions.

**Table 5:** The K_b_ values of the RUPA-BSA system with and without metal ions

System	Log K_b_	K_b_ (M^− 1^)	*R* ^2^	SEM
BSA+RUPA	2.55	0.35 × 10^3^	0.9993	0.002
BSA+RUPA + Na^+1^	2.27	0.19 × 10^3^	0.9983	0.005
BSA+RUPA + K^+1^	3.74	5.50 × 10^3^	0.9993	0.004
BSA+RUPA + Mg^+2^	3.28	1.91 × 10^3^	0.9992	0.003
BSA+RUPA + Ca^+2^	4.15	14.13 × 10^3^	0.9996	0.005
BSA+RUPA + Zn^+2^	3.77	5.89 × 10^3^	0.9993	0.003
BSA+RUPA + Fe^+3^	2.58	0.38 × 10^3^	0.9999	0.001

*SEM standard error of mean

### Molecular docking analysis

To provide a comprehensive understanding of the various binding patterns, molecular modelling is mandatory, so a docking study of the RUPA into the BSA protein was conducted. The most popular model serum albumin protein for researching these kinds of protein-ligand interactions is BSA [38]. To conduct the analysis, the protein structure (PDB code 4f5s) was selected from the Protein Data Bank using MOE 2024.06 [39].

#### Conformational search

Conformational analysis of RUPA has been obtained for accurate further modeling studies, where its best and least energy conformer was produced by conformational search procedures that explore conformational space in torsional space, applying the multi-conformer approach. The results have been depicted in Figure S4.

#### Molecular docking and surface mapping

As illustrated in Figure S5 (a, b), the following amino acid residues were discovered to surround RUPA: Try149, Ser286, Arg256, Leu237, Ala290, Arg194, Arg217, Glu291, Lys187, and Asp450. The residues of the interacting amino acids are nearly identical with a small variation and are located in a hydrophobic cavity at site I. Since no hydrogen bonding contacts were found, it can be assumed that RUPA’s interaction with Try149 and Arg217 is solely hydrophobic via arene cationic interaction.

It was worth mentioning that BSA contains a second tryptophan residue Trp-134 located in subdomain IB (sometimes referred to as site III), in addition to the well-known Trp-213 in subdomain IIA (site I), however herein RUPA docking studies have shown from the surrounding amino acid residues that it fits well within site I, locating into the hydrophobic pocket of subdomain IIA in the vicinity of Tryptophan residues, specifically Trp213 with a calculated binding energy of −6.41 kcal/mol which is consistent with experimental data (Figure S5 (c)).

Numerous studies have demonstrated how hydrophobic interactions contribute to the stability of drug-serum albumin complexes [40], which encouraged us to conduct an in-depth surface mapping investigation.

Further study about the relation between the hydrophobic character of RUPA and the existence of residues of hydrophobic amino acids at the BSA binding site has shown that this similarity may have helped to stabilize the RUPA-BSA system via hydrophobic forces; that was basically confirmed by both observing the 3D view of the overlay of RUPA into the binding pocket of BSA as revealed in Figure S5 (b) and the surface map calculation of RUPA (Figure S6) showing a total green color confirming its hydrophobic character. The primary role of these hydrophobic forces is to energetically sustain RUPA at the BSA interface.

## Limitations of the study

This study was performed in vitro, utilizing integrated spectroscopic techniques (fluorescence spectroscopy, ultraviolet-visible spectroscopy, and FTIR) alongside molecular docking to examine the interaction between RUPA and BSA. Spectroscopic studies yield indirect evidence of binding and structural alterations, whereas the docking data serve as predictive computational models that do not entirely mimic physiological settings. Furthermore, only bovine serum albumin was tested, and interactions with other serum proteins or in vivo systems were not considered. As a result, additional experimental and biological validation studies are required to confirm and expand these findings.

## Conclusions

In summary, fluorescence spectroscopy, ultraviolet-visible spectroscopy, FTIR, and docking were employed to investigate the biomolecular binding behavior of the RUPA-BSA interaction. According to the ultraviolet-visible spectra and the fluorescence study, BSA and RUPA can bind together to generate a ground-state complex through a static quenching mechanism. The determined order of the binding constant for the RUPA-BSA is 10^3^ L mol^− 1^, indicating weak to moderate affinity for binding. FTIR data obtained for the RUPA-BSA complex suggest that the secondary structure of the protein undergoes alterations during the interaction. According to the positive values of the thermodynamic characteristics (∆H° and ∆S°), hydrophobic forces stabilized the RUPA-BSA complex system. As well, the negative ΔG values in the temperature range under study suggest that the binding process occurs spontaneously. The docking analysis further corroborated the findings of the site probing experiment that site I is the binding site for RUPA at BSA, in the vicinity of tryptophan residues, and primarily by hydrophobic interactions. Docking study indicated that the ideal RUPA conformer has the lowest binding energy in the IIA subdomain of the site. I. These findings are in line with those obtained from the site marker assay and the synchronous fluorescence measurements.

## Supplementary Information

Supplementary Material 1

## Data Availability

This work did not generate any new macromolecular structures or other datasets requested by the repository. The three-dimensional structure of bovine serum albumin utilized for molecular modeling was obtained from the Protein Data Bank (PDB) (PDB code 4f5s). The datasets used and/or analyzed during the current study are available from the corresponding author on reasonable request.
