# Supplementary material for: Exploring the binding interaction of rupatadine with bovine serum albumin using multi-spectroscopic and molecular modeling approaches
Source: BMC Chem. 2026 Mar 28;20(1):81. doi: 10.1186/s13065-026-01769-2 (PMC13064039; doi:10.1186/s13065-026-01769-2)
Supplement: Supplementary file 1 — Supplementary Material 1 [file 13065_2026_1769_MOESM1_ESM.docx]

**Exploring the binding interaction of rupatadine with bovine serum albumin using multi-spectroscopic and molecular modeling approaches**

**Heba Abo Shamiya^1^, Heba Elmansi^1^, Shahenda M. El-messery^2^, Fathalla Belal^1^**

^1^ Department of Pharmaceutical Analytical Chemistry, Faculty of Pharmacy, Mansoura University, 35516 Mansoura, Egypt

^2^ Department of Pharmaceutical Organic Chemistry, Faculty of Pharmacy, Mansoura University, 35516 Mansoura, Egypt

Tel.: ++20502247496 Fax: ++20502247496 e-mail: [boshamiyaheba@gmail.com](mailto:boshamiyaheba@gmail.com)

Supplementary Figures:

Figure S1: The Modified Stern-Volmer plot for BSA-RUPA at various temperatures.

Figure S2: Inner-filter-corrected synchronous fluorescence of BSA-RUPA complex at ∆λ=15 (a) and ∆λ=60 (b) with increasing the concentration of RUPA (0.00-100µM) and 2µM of BSA at pH 7.4 at 303 K.

Figure S3: (a) Free BSA FTIR spectra at 303 K at pH 7.4.

(b) BSA-RUPA complex FTIR spectra at 303 K at pH 7.4.

Figure S4: Lowest energy conformer of compound Rupatadine with balls and cylinders rendering.

Figure S5: (a) The 2D binding mode and residues involved in the recognition of rupatadine

(b) 3D binding view of rupatadine embedded into the surface mapping pocket. Pink: hydrogen bond, blue: mild polar, green: hydrophobic region.

(C) 3D rupatadine view illustrating that binding occurs near the Trp213 residue

Figure S6: Surface map for Rupatadine, Pink: hydrogen bond, blue: mild polar, green: hydrophobic region.

Figure S7: UV absorption spectra of rupatadine (50µM).

Figure S8: Apparent molar absorptivity spectra of BSA (2μM) in the presence of varying concentrations of RUPA (0–100μM) at pH 7.4 and 303 K.

**
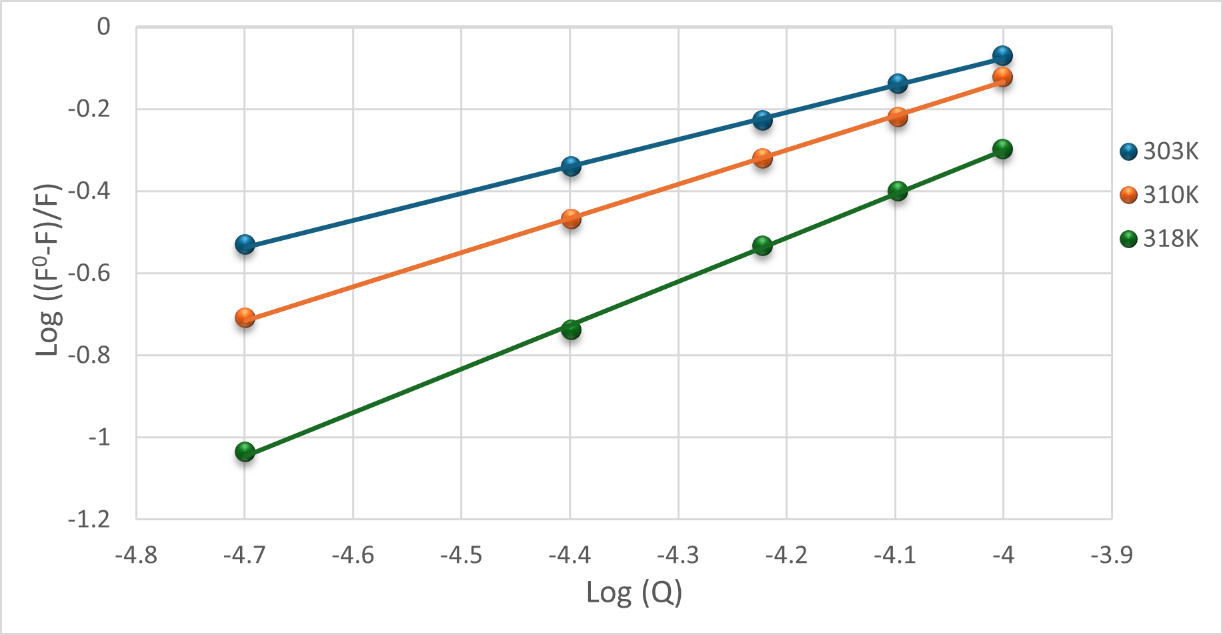
**

**Figure S1**

**
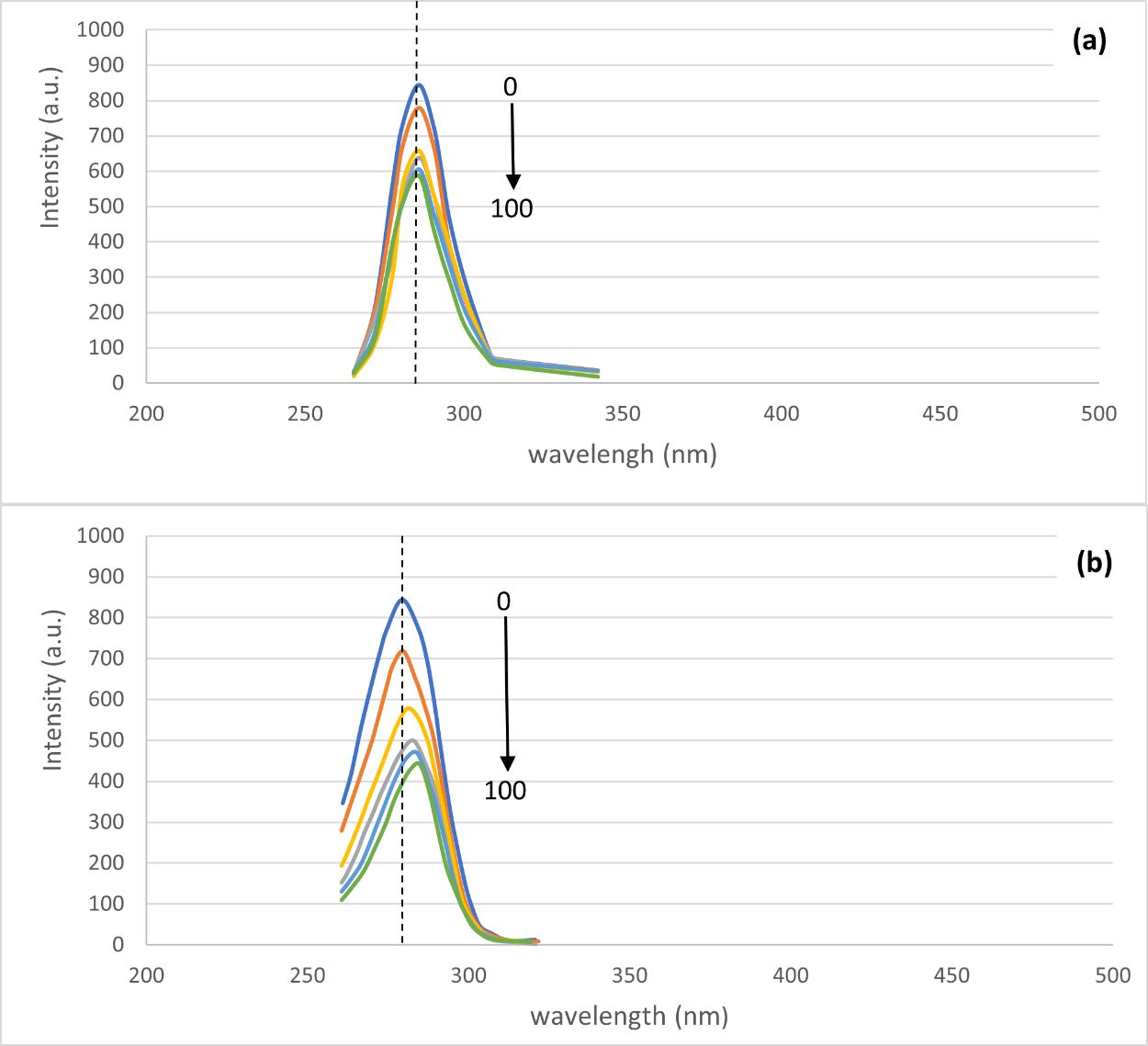
**

**Figure S2**

**
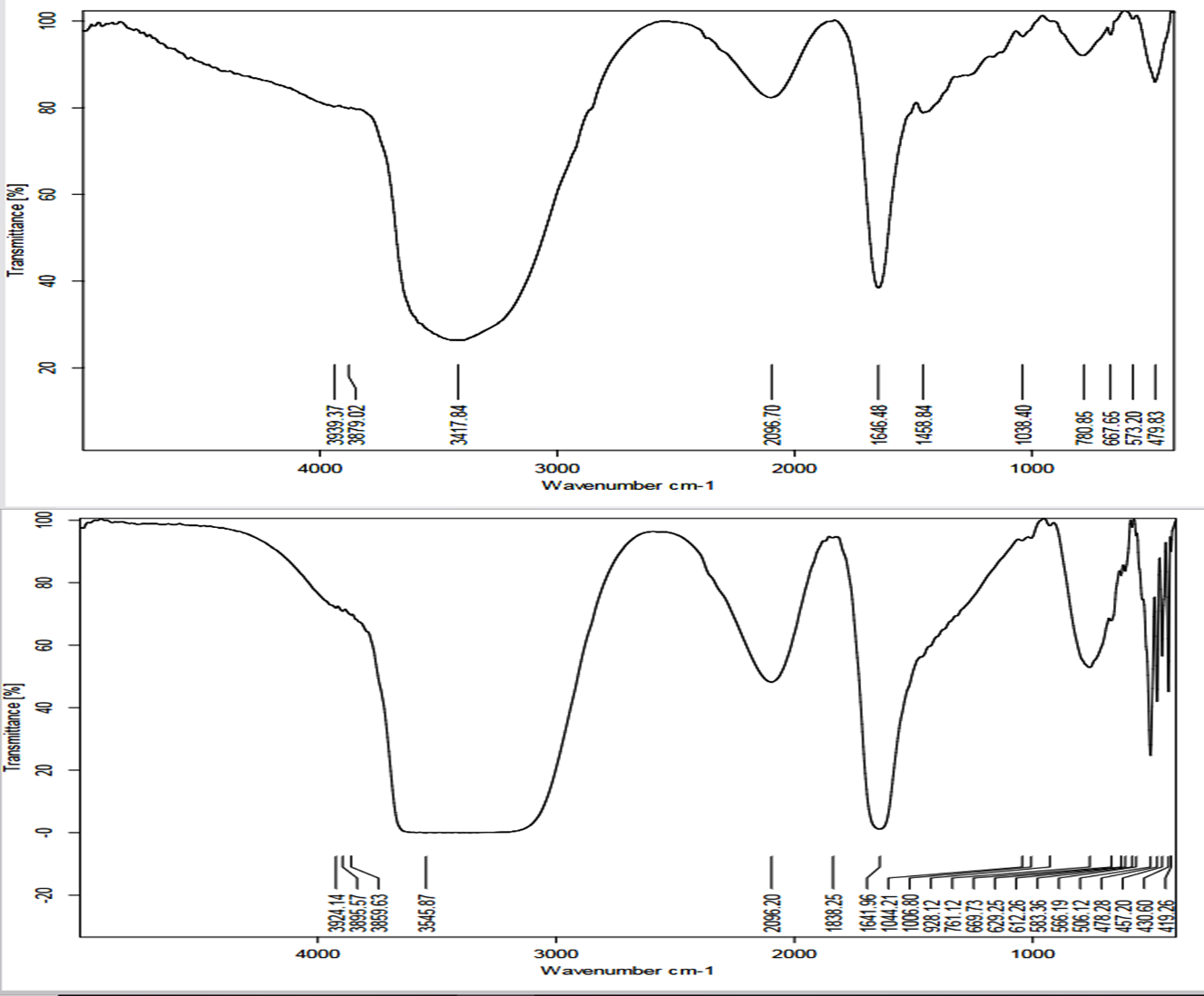
**

**Figure S3**


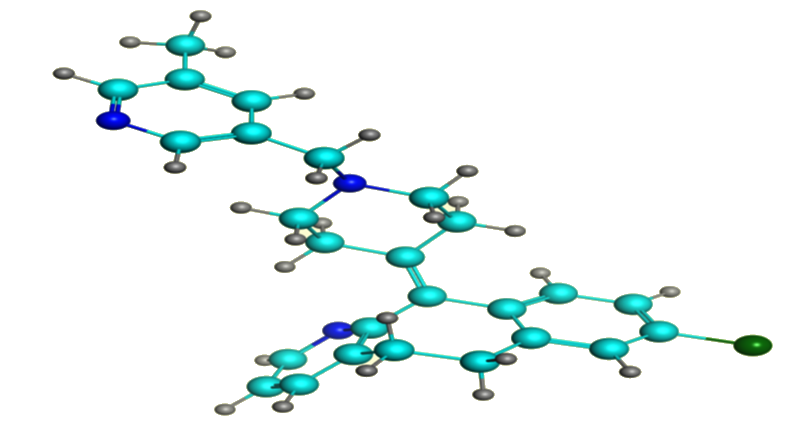


**Figure S4**


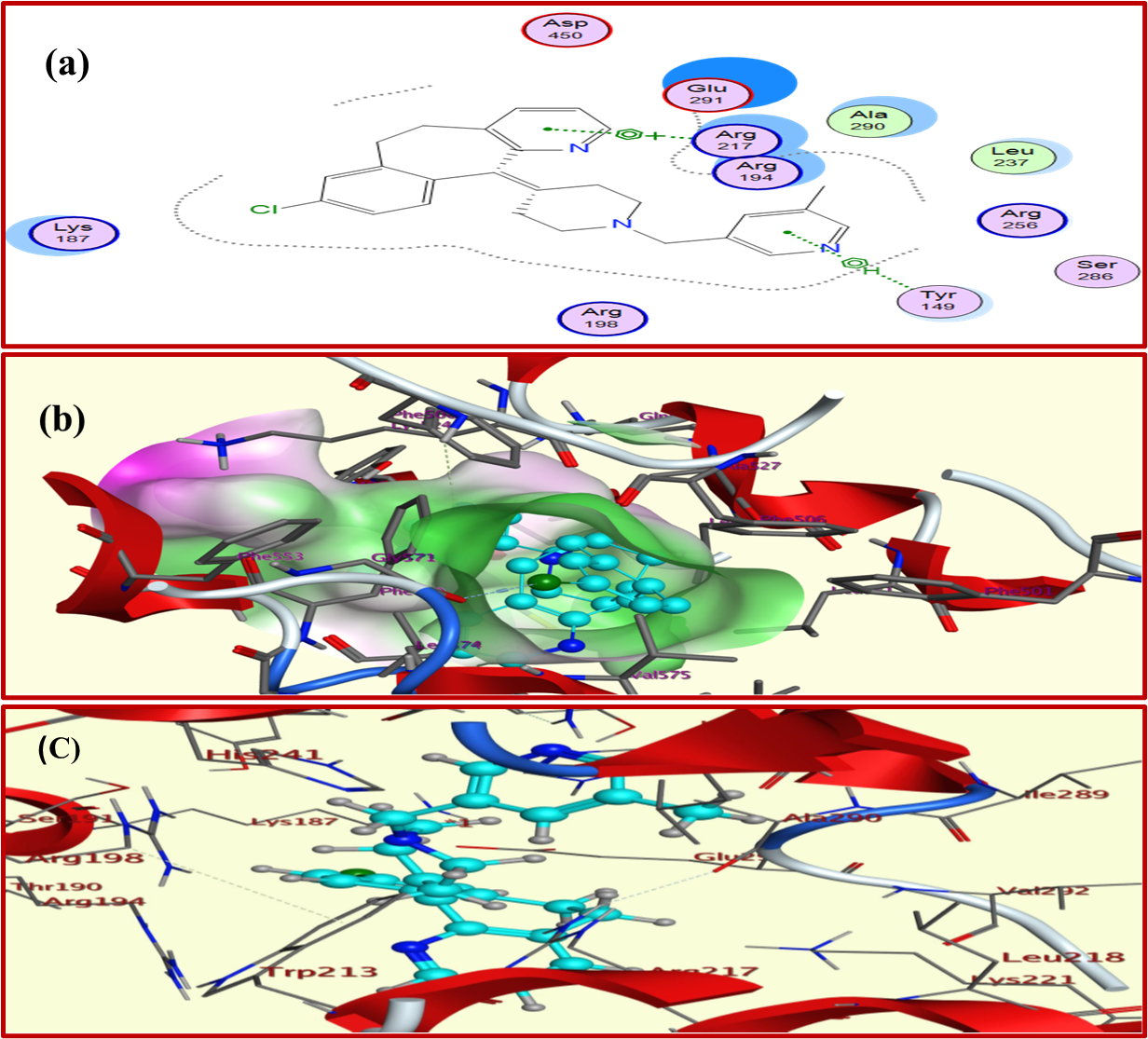


**Figure S5**


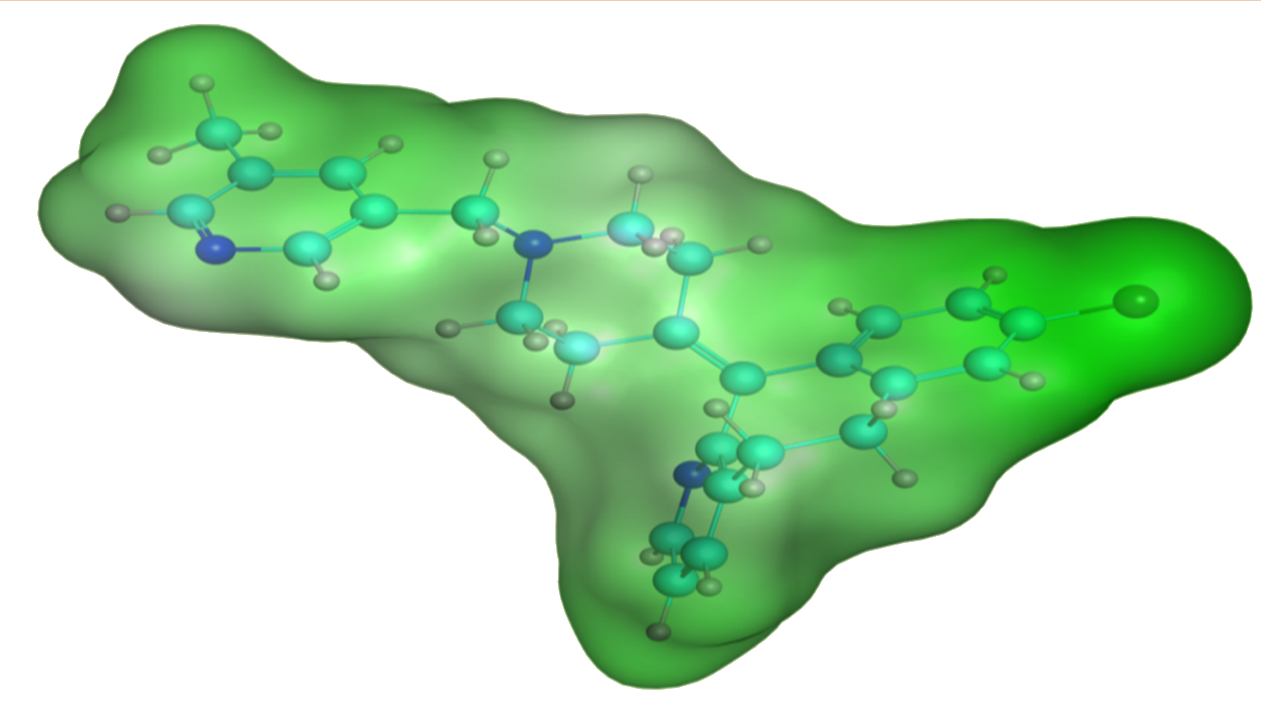


**Figure S6**

**
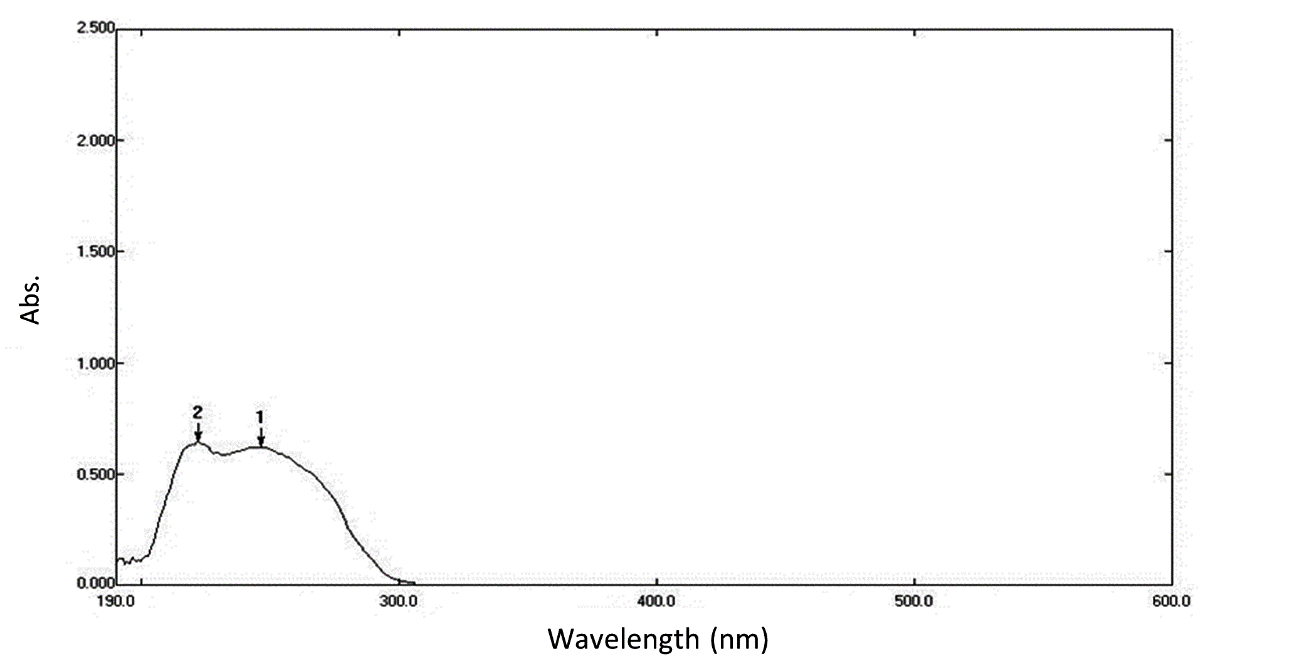
**

**Figure S7**


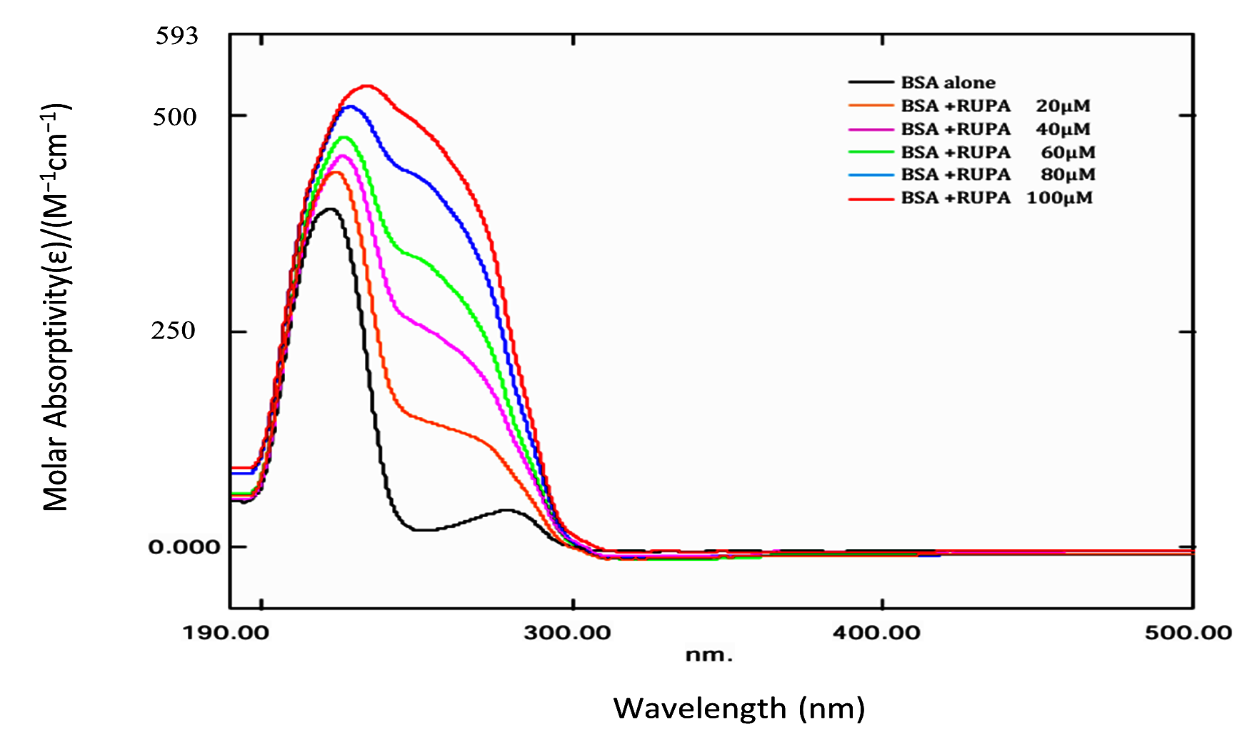


**Figure S8**
